# Global leaders malnutrition initiative-defined malnutrition affects long-term survival of different subgroups of patients with gastric cancer: A propensity score-matched analysis

**DOI:** 10.3389/fnut.2022.995295

**Published:** 2022-09-30

**Authors:** Wentao Cai, Hui Yang, Jingwei Zheng, Jianqiang Huang, Weiping Ji, Yangbin Lu, Xinxin Yang, Weiteng Zhang, Xian Shen, Xiaodong Chen

**Affiliations:** ^1^Department of Gastrointestinal Surgery, The First Affiliated Hospital, Wenzhou Medical University, Wenzhou, China; ^2^Department of Gastrointestinal Surgery, The Second Affiliated Hospital, Wenzhou Medical University, Wenzhou, China

**Keywords:** GLIM, gastric cancer, overall survival, subgroups, malnutrition

## Abstract

As defined by the Global Leaders Malnutrition Initiative (GLIM), malnutrition is strongly associated with a lower quality of life and poor prognosis in gastric cancer patients. However, few studies have precisely explored the predictors of malnutrition, as defined by the GLIM, for overall survival (OS) after gastric cancer surgery in subgroups of patients stratified according to population characteristics. Our research aimed to analyze whether the predictors of malnutrition defined by the GLIM for postoperative OS in gastric cancer patients differ across subgroups. Patients who underwent radical gastric cancer surgery at our center between July 2014 and February 2019 were included in the study. Propensity score matching (PSM) was used to minimize bias. The study population was divided into malnourished and normal groups based on whether they were malnourished as defined by the GLIM. Univariate and multivariate analyses were performed to identify the risk factors affecting OS. The Kaplan–Meier curve and log-rank test were performed to determine the survival rate difference between subgroups. Overall, 1,007 patients were enrolled in the research. Multivariate analysis showed that malnutrition among the patients was 33.47%. Additionally, GLIM-defined malnutrition was an independent risk factor [hazard ratio (HR): 1.429, *P* = 0.001] for a shorter OS in gastric cancer patients. Furthermore, subgroup analysis showed that the GLIM was more appropriate for predicting OS in older aged patients (≥65 years), females, those with comorbidities (Charlson comorbidity index ≥ 2), and those with advanced gastric cancer (TNM stage = 3). GLIM-defined malnutrition affects the long-term survival of gastric cancer patients, especially older patients, females, patients with comorbidities, and patients with advanced gastric cancer.

## Introduction

Gastric cancer has the fifth highest incidence among cancers and is the third most common cause of cancer-related death. Every year, at least 1 million new cases are diagnosed worldwide, most of which are in Asia, Eastern Europe, and South America (1). Currently, surgical resection remains the most effective treatment (2). Although gastric cancer incidence and mortality rates have decreased, the mortality rate still reaches 75% (3), which places a significant burden on the economy, society, and the patient’s family. Hence, it is critical to anticipate and improve factors that reduce the survival rates of gastric cancer patients after surgery to improve outcomes. Malnutrition has been a major issue in international health care and is not only associated with a poor prognosis but also results in higher rates of infection and complications (4), prolonged hospital stays, and increased mortality (5, 6). Malnutrition is more prevalent in cancer patients, especially older age in some community hospitals (7); hence, its adverse effects result in more severe outcomes. Therefore, it is necessary to focus on the nutritional status of cancer patients (8). Previously, malnutrition awareness was low, resulting in improper management of malnutrition. Additionally, there was no general understanding of malnutrition’s definition, prevalence, and identification (9). The Global Leadership Initiative in Malnutrition (GLIM) was successfully held in 2016 to define a consensus on malnutrition. It proposes a diagnostic criterion for malnutrition that can be adapted to different clinical settings, is simple to implement and has global expert consensus. The result of the meeting is a two-step model for screening and assessment of malnutrition. Using these criteria, patients with malnutrition, especially the older aged, were found to have an increased risk of death during the community follow-up (10), affecting both the postoperative overall and disease-free survival in gastric cancer patients (11). However, no other research has reported GLIM-defined malnutrition’s predictive capability in subgroups of populations with different characteristics. Hence, we aimed to investigate whether the predictive value of GLIM-defined malnutrition differs in subgroups of populations and whether this definition may more accurately predict the individual overall survival (OS) of gastric cancer patients.

## Materials and methods

### Patients

The basic information of all patients who underwent radical gastrectomy in the First Affiliated Hospital of Wenzhou Medical University from July 2014 to February 2019 was collected retrospectively. The inclusion criteria were: (1) diagnosis of gastric adenocarcinoma confirmed by preoperative or postoperative pathology; (2) American Association of Anesthesiologists grade ≤ III; and (3) no distant metastasis. The exclusion criteria were: (1) patients who underwent palliative resection or emergency surgery; (2) patients who received chemotherapy before surgery; and (3) patients without basic clinical information or computed tomography (CT) data 1 month before surgery. All surgeries were performed by a single surgical team, thus avoiding possible bias caused by the effectiveness of surgical treatment. The research was performed in compliance with the Declaration of Helsinki. Ethical approval was obtained from the First Affiliated Hospital, Wenzhou Medical University. All participants provided written informed consent.

### Skeletal muscle index assessment

We used the image processing system (GE ADW 4.5) to process the patient’s CT images within the first month before surgery. We adjusted the Hounsfield unit threshold to -29 to +150 to differentiate skeletal muscle from other tissues. Then a trained investigator manually outlined the area of skeletal muscle at the third lumbar spine (L3) level, which includes the psoas major, erector spinae, quadratus lumborum, oblique abdominis, external and internal oblique muscles, and rectus abdominis. The outlined area of skeletal muscle at the L3 level normalized by height (m^2^) was used to obtain the skeletal muscle mass index (SMI).

### Global leaders malnutrition initiative assessment

As a two-step model for screening and diagnosing malnutrition, the first step of GLIM is to identify individuals who may be at potential risk of malnutrition through some internationally recognized malnutrition risk screening scales; here, we chose the Nutrition Risk Screening 2002 (12). The second step is to evaluate identified individuals and classify them according to their diagnosis and severity of malnutrition. The GLIM comprises phenotypic and etiological criteria. Malnutrition based on the GLIM must meet at least one phenotype combined with one etiological criterion. Phenotypic criteria include muscle mass loss, low BMI, and non-volitional weight loss. Etiological criteria include the reduction of food intake or assimilation, disease burden, or inflammation. Our target population comprised patients with gastric cancer. As cancer already meets the etiological standard of disease burden, malnutrition can be diagnosed as long as they meet a phenotypic criterion. Non-volitional weight loss is weight loss >5% within half a year or >10% beyond half a year. Low BMI is a BMI <20 and <18.5 kg/m^2^ for patients ≥70 and <70 years old, respectively (12). We chose the SMI of the L3 level as an index to assess muscle mass. According to our previous research, the critical value of SMI is 34.9 cm^2^/m^2^; for males, the value is 40.8 cm^2^/m^2^ (13). Therefore, a diagnosis of malnutrition can be made if our patients meet any of the above phenotypic criteria.

### Data collection

We retrospectively collected the clinical information of all patients who met the inclusion criteria in this study. The clinical data were divided into three categories: (1) basic clinical information before the operation, including age, sex, BMI, recent weight loss, preoperative CT images, serum albumin concentration (<35 g/L is considered hypoalbuminemia), American Society of Anesthesiologists grade, and Charlson comorbidity index (CCI); (2) surgery and tumor-related data, including operation method, type of resection, tumor differentiation, and TNM stage; and (3) postoperative clinical outcomes, including length of hospitalization, hospitalization costs, postoperative survival condition, and postoperative survival time. Experienced physicians obtained postoperative survival outcomes over the phone or on an outpatient basis. Telephone follow-ups were conducted every 3 months. Five years of follow-up or the patient’s death were considered the end of follow-up.

### Statistical analysis

Propensity score matching (PSM) was performed to reduce differences in clinical information between the GLIM-defined malnutrition group and the normal group. The matched factors differed between the two groups and affected the OS of patients (statistically significant factors in univariate regression analysis). We selected age, sex, hypoalbuminemia, operation method, differentiation, and TNM stage as matching factors to construct the PSM model based on the preliminary statistical results. We used a 1:2 ratio for matching with a matching precision of 0.05. All normally distributed continuous variables are expressed as a mean and standard deviation. Otherwise, they are expressed as median and interquartile ranges. The independent sample *t*-test and chi-square test (or Fisher’s exact test) were used to analyze the differences between continuous variables and classify variables between the two groups. The Kaplan–Meier curve and log-rank test were used to determine the survival difference between the groups. The proportional hazards model was used to determine the risk factors affecting survival. Factors that were statistically significant in the univariate analysis were included in the multivariate analysis to identify independent risk factors affecting the OS of patients. A double-tailed *P* < 0.05 was considered statistically significant. All statistical analyses were performed using IBM SPSS Statistics software (version 25.0; IBM Corp., Armonk, NY, USA).

## Results

### Patients

From July 2014 to February 2019, 1,007 eligible patients were enrolled. As shown in Table 1, there were 337 patients in the GLIM-defined malnutrition group and 670 patients in the normal group, with a malnutrition rate of 33.5%. For the population characteristics, the malnourished group had a higher proportion of women (*P* = 0.008), were older (*P* < 0.001), and had lower albumin levels (*P* < 0.001). For surgical selection and tumor information, the malnourished group preferred open surgery (*P* < 0.001), had less tumor differentiation (*P* < 0.001) and had a higher TNM stage (*P* < 0.001).

**TABLE 1:** Patient baseline characteristics.

Factors	Malnutrition (*n* = 337)	Normal (*n* = 670)	*P*
Sex			0.008*
Female	107 (31.80%)	160 (23.90%)	
Male	230 (68.20%)	510 (76.10%)	
Age, years			0.001*
<65	131 (38.90%)	332 (49.60%)	
≥65	206 (61.10%)	338 (50.40%)	
Hypoalbuminemia			<0.001*
No	221 (65.60%)	537 (80.10%)	
Yes	116 (34.40%)	133 (19.90%)	
Charlson comorbidity index			0.447
≤1	286 (84.90%)	556 (83.00%)	
≥2	51 (15.10%)	114 (17.00%)	
Operation method			<0.001*
Open	243 (72.10%)	408 (60.90%)	
Laparoscopy	94 (27.90%)	262 (39.10%)	
Type of resection			0.102
Subtotal gastrectomy	199 (59.10%)	431 (64.30%)	
Total gastrectomy	138 (40.90%)	239 (35.70%)	
Differentiation			<0.001*
High/Middle	79 (23.40%)	236 (35.20%)	
Low	258 (76.60%)	434 (64.80%)	
TNM stage			<0.001*
	62 (18.40%)	286 (42.70%)	
	82 (24.30%)	135 (20.10%)	
	193 (57.30%)	249 (37.20%)	
Length of hospitalization	14.00 (8)	13.00 (6)	0.004*
Hospitalization costs	63166.13 (23444.79)	57010.58 (20629.87)	<0.001*

*Statistically significant (*P* < 0.05, two-tailed).

### Univariate and multivariate analyses related to survival outcomes

Table 2 shows that in the univariate analysis, age, GLIM-defined malnutrition, hypoalbuminemia, laparoscopic surgery, total gastrectomy, tumor differentiation, and TNM stage all affected OS after surgery. As the three phenotypic criteria of GLIM, low BMI, weight loss and low SMI were also significantly associated with the survival of gastric patients. In the multivariate analysis, considering the large correlation between these three phenotypic criteria and GLIM, if they and GLIM were included in the multivariate analysis at the same time, it would cause unavoidable bias to the results, so we did not include them in the multivariate analysis, after adjusting for TNM stage, age, hypoalbuminemia, laparoscopic surgery, total gastrectomy, and tumor differentiation, GLIM-defined malnutrition was revealed to be an independent risk factor for postoperative OS [hazard ratio (HR): 1.429, *P* = 0.001]. Similarly, age (HR: 1.726, *P* < 0.001), total gastrectomy (HR: 1.450, *P* < 0.001), tumor differentiation (HR: 1.504, *P* = 0.002), and TNM stage (II/I HR: 2.162, *P* < 0.001; III/I HR: 5.738, *P* < 0.001) were independent risk factors for postoperative OS in gastric cancer patients.

**TABLE 2:** Univariate and multivariate Cox regression analysis of factors associated with overall.

	Univariate analysis		Multivariate analysis	
Factors	HR (95% CI)	*P*	HR (95% CI)	*P*
**Age, years**				
<65	Ref		Ref	
≥65	1.831 (1478–2.268)	<0.001*	1.726 (1.385–2.151)	<0.001*
**SEX**				
Female	Ref			
Male	1.274 (0.999–1.625)	0.051		
**BMI**				
Normal	Ref			
Low	1.859 (1.475–2.342)	<0.001*		
**Weight loss**				
No	Ref			
yes	2.064 (1.657–2.572)	<0.001*		
**SMI**				
Normal	Ref			
Low	1.718 (1.352–2.184)	<0.001*		
**Malnutrition**				
Normal	Ref		Ref	
Defined by GLIM	2.085 (1.699–2.559)	<0.001*	1.429 (1.159–1.762)	0.001*
**Charlson comordity index**				
≤1	Ref			
≥2	1.090 (0.831–1.429)	0.534		
Hypoalbuminemia				
No	Ref		Ref	
Yes	1.868(1.508–2.314)	<0.001*	1.148 (0.920–1.433)	0.222
**Operation method**				
Open	Ref		Ref	
Laparoscopy	0.552 (0.435–0.700)	<0.001*	0.846 (0.664–1.079)	0.178
**Type of resection**				
Subtotal gastrectomy	Ref		Ref	
Total gastrectomy	2.034 (1.658–2.495)	<0.001*	1.450 (1.178–1.784)	< 0.001*
**Differentiation**				
High/middle	Ref		Ref	
Low	2.165 (1.678–2.794)	<0.001*	1.504 (1.160–1.951)	0.002*
**TNM stage**				
I	Ref		Ref	
II	2.934 (1.962–4.389)	<0.001*	2.162 (1.432–3.263)	<0.001*
III	8.071 (5.742–11.344)	<0.001*	5.738 (4.009–8.215)	<0.001*

*Statistically significant (*P* < 0.05, two-tailed).

### Propensity score matching and subgroup analysis based on population characteristics

**FIGURE 1:**
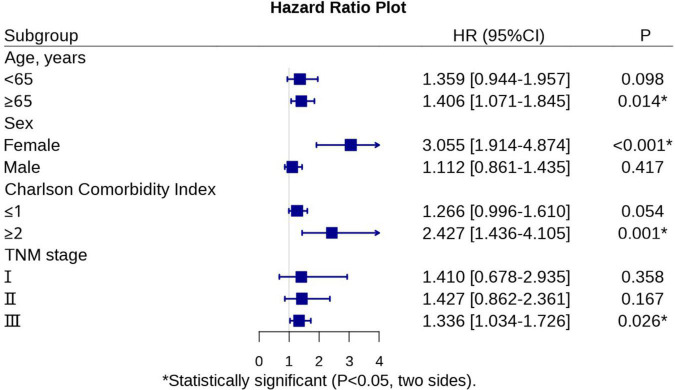
Hazrad ratio plot of subgroups analysis.

**FIGURE 2:**
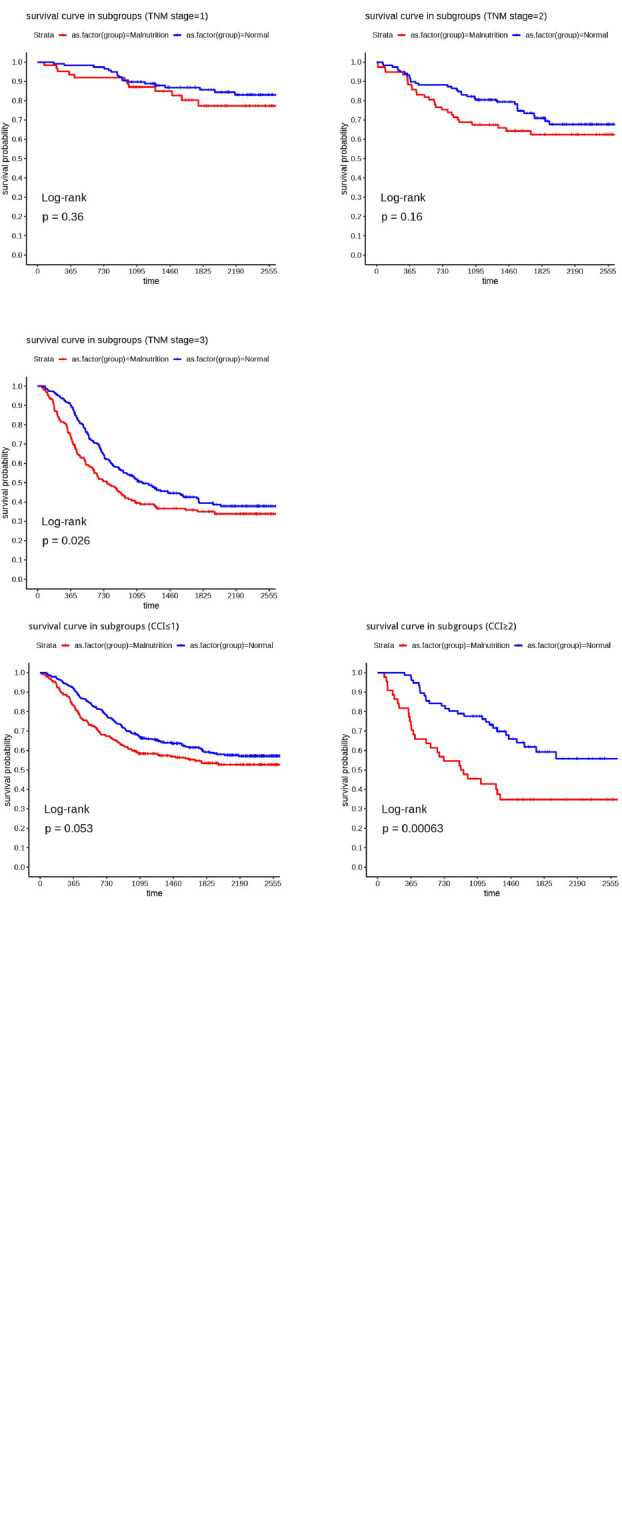
The Kaplan–Meier curve of subgroups.

Matching factors were included as described previously. We selected age, sex, hypoalbuminemia, lumpectomy, tumor differentiation, and TNM stage as matching conditions, after matching, the total number of patients was reduced from 1007 to 764, including 301 in the malnutrition group and 463 in the normal group. There was no statistical discrepancy in the basic clinical information between the two groups, as shown in Table 3. After PSM, subgroup analyses showed that malnutrition defined by the GLIM had a better predictive capability for OS in gastric cancer patients in the following subgroups: aged ≥ 65 years (HR: 1.406, *P* = 0.014); females (HR: 3.055, *P* < 0.001); CCI ≥ 2 (HR: 2.427, *P* = 0.001); and TNM stage 3 (HR: 1.336, *P* = 0.026) (Table 4). We have created Figure 1 to represent the subgroup analysis results clearly. Figure 2 show the differences in survival curves between the malnourished and normal groups in the different subgroups. Survival was lower in the malnourished group among those aged ≥65 years, whereas in those aged <65 years, there was no statistical discrepancy in survival between the two groups. Correspondingly, in females and those with a CCI ≥ 2 and TNM stage 3, survival was lower in the malnourished group than in the normal group, as shown in Table 4.

**TABLE 3:** Patient baseline characteristics after PSM.

Factors	Malnutrition (*n* = 301)	Normal (*n* = 463)	*P*
Gender			0.065
Female	100 (33.20%)	125 (27.00%)	
Male	201 (66.80%)	338 (73.00%)	
Age, years			0.297
<65	125 (41.50%)	210 (45.40%)	
≥65	176 (58.50%)	253 (54.60%)	
Hypoalbuminemia			0.418
No	219 (72.80%)	349 (75.40%)	
Yes	82 (27.20%)	114 (24.60%)	
Charlson comorbidity index			0.505
≤1	257 (85.40%)	387 (83.60%)	
≥2	44 (14.60%)	76 (16.40%)	
Operation method			0.308
Open	214 (71.10%)	313 (67.60%)	
Laparoscopy	87 (28.90%)	150 (32.40%)	
Type of resection			0.763
Subtotal gastrectomy	182 (60.50%)	285 (61.60%)	
Total gastrectomy	119 (39.50%)	178 (38.40%)	
Differentiation			0.605
High/Middle	73 (24.30%)	120 (25.90%)	
Low	228 (75.70%)	343 (74.10%)	
TNM stage			0.294
I	62 (20.60%)	117 (25.30%)	
II	77 (25.60%)	118 (25.50%)	
III	162 (53.80%)	228 (49.20%)	
Length of hospitalization	14.00 (8)	13.00 (7)	0.178
Hospitalization costs	62890.34 (23048.06)	57249.26 (21104.70)	0.004*

*Statistically significant (*P* < 0.05, two sides).

**TABLE 4:** Univariate analysis of GLIM-defined malnutrition on overall survival in subgroups.

Sub-group	Univariate analysis	
	HR (95% CI)	*P*
**Age, years**		
<65	1.359 (0.944–1.957)	0.098
≥65	1.406 (1.071–1.845)	0.014*
**Sex**		
Female	3.055 (1.914–4.874)	<0.001*
Male	1.112 (0.861–1.435)	0.417
**Charlson comorbidity index**		
≤1	1.266 (0.996–1.610)	0.054
≥2	2.427 (1.436–4.105)	0.001*
**TNM stage**		
	1.410 (0.678–2.935)	0.358
	1.427 (0.862–2.361)	0.167
	1.336 (1.034–1.726)	0.026*

*Statistically significant (*P* < 0.05, two sides).

## Discussion

Depending on the diagnostic criteria, the prevalence of GLIM-defined malnutrition ranged from 19 to 48.4% in different populations (14–16). In our study, 1,007 patients were enrolled, of which 337 (33.47%) were malnourished, as defined by the GLIM. Multivariate analysis in this study showed that malnutrition, defined by the GLIM, was an independent risk factor for OS in gastric cancer patients who underwent surgical treatment. Several studies have pointed out that malnutrition has a considerable negative effect on the OS of cancer patients. Zhang et al. showed that malnutrition, identified by comprehensive geriatric assessment, increased all-cause mortality in older patients diagnosed with solid tumors (6). Li et al. showed that, based on the midarm circumference or hand grip strength, severe malnutrition defined by the GLIM increases the risk of death in gastric cancer patients (17). Huang et al. pointed out that malnutrition, defined by GLIM based on the SMI obtained from abdominal CT images, affects the survival time of gastric cancer patients (18). All these results are consistent with ours. Therefore, identifying malnutrition and early nutritional intervention is critical to prolonging the survival of gastric cancer patients.

This study mainly aimed to explore the characteristics of populations suitable for GLIM assessment of malnutrition to predict OS more accurately. The differences in characteristics between the malnourished and normal groups converged after PSM, leading to more robust results after univariate analysis. In the subgroup analysis, malnutrition defined by the GLIM had a better predictive capability for OS in patients aged ≥65 years, females, patients with CCI ≥ 2, and patients with progressive gastric cancer (TNM stage = 3).

Age is an independent risk factor for the prognosis of patients with cancer, and this has been confirmed in many studies, a study by Xu et al. (19) found that age ≥70 years was an independent risk factor in postoperative gastric cancer patients. Of the patients included in this study, 61.10 and 50.40% of patients in the malnourished and normal groups were older (aged ≥65 years), indicating that this subgroup may be at greater risk for malnutrition. Rodríguez-Mañas et al. (20) also showed that malnourished people are often older and in worse physical condition. Therefore, older patients are at greater risk of malnutrition and have a poorer physiological profile. Hence, malnourishment before surgery is less likely to be corrected after surgery, resulting in shorter survival. In contrast, younger patients’ overall nutritional and physiological status is better. Thus, even if malnutrition is diagnosed preoperatively, it can be corrected postoperatively with appropriate interventions. Therefore, preoperative malnutrition may not accurately predict postoperative OS in the younger population.

Keaver et al. noted that women with cancer are at a higher risk of malnutrition and are more likely to reach the clinical significance thresholds for quality-of-life subscales, such as physical functioning, fatigue, and pain, compared to men (21). Park et al. showed that female is an independent risk factor for malnutrition within 6 months after gastrectomy (22). Therefore, the susceptibility to malnutrition among female patients may make it difficult for clinicians to correct malnutrition after gastric cancer surgery, leading to the shorter survival of malnourished female patients.

The CCI is an objective quantification of comorbidity, and it has been shown that a CCI ≥ 2 shortened OS by 3 years in patients with esophageal cancer (23). In our research, we used an age-adjusted CCI, where patients with a CCI ≥ 2 and a higher risk of malnutrition (as defined by GLIM) exhibited low OS. However, the difference in survival between the malnourished and normal groups having a CCI ≤ 1 was not observed. The CCI consists of scores for circulatory disorders, digestive disorders, and other malignancies. We hypothesize that when malnutrition is combined with these disorders, it can cause severe damage to the patient’s physiological metabolism and body functions, leading to an increased risk of death.

It is well known that the TNM stage significantly impacts the prognosis of patients; the higher the stage, the worse the survival (24). Our results showed that GLIM-defined malnutrition shortened the postoperative OS of gastric cancer patients beginning at TNM stage 3. Therefore, we hypothesize that the physical status, mental health, and overall quality of life of patients with advanced gastric cancer are reduced. Furthermore, the interaction between malnutrition and disease may lead to more pronounced physiological decline and accelerate tumor progression, further contributing to shorter OS in malnourished patients.

For the present, this study is the first to assess the difference in predictive value of malnutrition defined by GLIM for patient OS across different subgroups of the population. However, the study has some limitations. Firstly, this is a retrospective study, and further prospective trials are needed to validate it in the future further. Secondly, all patients in this study were from the same center, which may result in selection bias. Finally, the cut-off values for muscle mass reduction defined in this study were derived from our previous large sample study. Whether this applies to other regional populations needs to be validated in further studies.

This study summarizes the differences in the effects of GLIM-defined malnutrition in different subgroups of the population. This can guide clinicians in treating gastric cancer patients, especially older women and those with comorbidities and advanced tumors. These patients may need to focus on preoperative and postoperative nutritional interventions to improve their malnutrition status as much as possible, thus effectively improving their long-term survival rate.

In conclusion, GLIM-defined malnutrition is an independent risk factor for OS in patients with gastric cancer. However, its predictive value is more advantageous in older patients, females, patients with comorbidities, and patients with advanced tumor stage.

## Data availability statement

The raw data supporting the conclusions of this article will be made available by the authors, without undue reservation.

## Ethics statement

The studies involving human participants were reviewed and approved by the Ethics Committee of the First Affiliated Hospital of Wenzhou Medical University. The patients/participants provided their written informed consent to participate in this study. Written informed consent was obtained from the individual(s) for the publication of any potentially identifiable images or data included in this article.

## Author contributions

WZ, XS, and XC were main manuscript authors and had contributed substantially to the study’s conception and design and gave final approval and revised the manuscript. HY, JH, YL, and JZ were involved in the data collection. XY and WJ were responsible for the data analysis and revision of the manuscript. WC wrote this manuscript. All authors have read and approved the final manuscript.

## Abbreviations

BMI: body mass index
CCI: Charlson comorbidity index
GLIM: Global Leaders Malnutrition Initiative
OS: overall survival
PSM: Propensity score matching
SMI: skeletal muscle index.
